# Supplementary material for: Modelling the concentration of anti-SARS-CoV-2 immunoglobulin G in intravenous immunoglobulin product batches
Source: PLoS One. 2021 Nov 29;16(11):e0259731. doi: 10.1371/journal.pone.0259731 (PMC8629175; doi:10.1371/journal.pone.0259731)
Supplement: S1 Table — (DOCX) [file pone.0259731.s001.docx]

*Modelling the concentration of anti-SARS-CoV-2 immunoglobulin G in intravenous immunoglobulin product batches.*

**Supplementary data**

**S1 Table – Mean anti-SARS-CoV-2 spike antibody concentration for each donor group, derived from Krammer et al. [3]**

| **Group** | **Infection Status** | **# Vaccine doses** | **Vaccination timepoint (weeks)** | | | | |
| --- | --- | --- | --- | --- | --- | --- | --- |
|  |  |  |  | **0** | **1** | **2** | **3** |
| **1** | Naive | 0 | **Anti-SARS-CoV-2 Ab concentration (AUC)** | 1 | 1 | 1 | 1 |
| **2** |  | 1 |  | 1 | 1 | 1016 | 1293 |
| **3** |  | 2 |  | 1293 | 1293 | 2606 | 3316 |
| **4** | Infected | 0 |  | 90 | 90 | 90 | 90 |
| **5** |  | 1 |  | 90 | 14208 | 25927 | 19534 |
| **6** |  | 2 |  | 19534 | 19534 | 29876 | 22509 |

Ab, antibody; AUC, area under the curve; SARS-CoV-2, severe acute respiratory syndrome coronavirus 2.
