# Supplementary material for: Modelling the concentration of anti-SARS-CoV-2 immunoglobulin G in intravenous immunoglobulin product batches
Source: PLoS One. 2021 Nov 29;16(11):e0259731. doi: 10.1371/journal.pone.0259731 (PMC8629175; doi:10.1371/journal.pone.0259731)
Supplement: S2 Table — (DOCX) [file pone.0259731.s002.docx]

*Modelling the concentration of anti-SARS-CoV-2 immunoglobulin G in intravenous immunoglobulin product batches.*

**Supplementary data**

**S2 Table.** **Anti-SARS-CoV-2 half-lives utilised for each donor group**

| **Group** | **Infection Status** | **# Vaccine doses** | **Half-life (days)** | **Reference** |
| --- | --- | --- | --- | --- |
| 1 | Naive | 0 | n/a |  |
| 2 |  | 1 | 58.5 | Doria-Rose *et al.*[11] *;* Khoury *et al.*[21] |
| 3 |  | 2 | 58.5 | Doria-Rose *et al.*[11] *;* Khoury *et al.*[21] |
| 4 | Infected | 0 | 20.4 | Barnes *et al.*[6] |
| 5 |  | 1 | 58.5 | Doria-Rose *et al.*[11] *;* Khoury *et al.*[21] |
| 6 |  | 2 | 58.5 | Doria-Rose *et al.*[11] *;* Khoury *et al.*[21] |

n/a, not available; SARS-CoV-2, severe acute respiratory syndrome coronavirus 2.
