# Supplementary material for: Modelling the concentration of anti-SARS-CoV-2 immunoglobulin G in intravenous immunoglobulin product batches
Source: PLoS One. 2021 Nov 29;16(11):e0259731. doi: 10.1371/journal.pone.0259731 (PMC8629175; doi:10.1371/journal.pone.0259731)
Supplement: S3 Table — (DOCX) [file pone.0259731.s003.docx]

*Modelling the concentration of anti-SARS-CoV-2 immunoglobulin G in intravenous immunoglobulin product batches.*

**Supplementary data**

**S3 Table.** **Relative proportion (in percent) of donors in each group per week (derived from data obtained from US CDC).**

| **Date** | **Group 1** | **Group 2** | **Group 3** | **Group 4** | **Group 5** | **Group 6** | **Total** |
| --- | --- | --- | --- | --- | --- | --- | --- |
| 05. Oct. 20 | 97.7 | 0 | 0 | 2.3 | 0 | 0 | 100 |
| 12. Oct. 20 | 97.6 | 0 | 0 | 2.4 | 0 | 0 | 100 |
| 19. Oct. 20 | 97.4 | 0 | 0 | 2.6 | 0 | 0 | 100 |
| 26. Oct. 20 | 97.3 | 0 | 0 | 2.7 | 0 | 0 | 100 |
| 02. Nov 20 | 97.1 | 0 | 0 | 2.9 | 0 | 0 | 100 |
| 09. Nov 20 | 96.9 | 0 | 0 | 3.1 | 0 | 0 | 100 |
| 16. Nov 20 | 96.5 | 0 | 0 | 3.5 | 0 | 0 | 100 |
| 23. Nov 20 | 96.1 | 0 | 0 | 3.9 | 0 | 0 | 100 |
| 30. Nov 20 | 95.8 | 0 | 0 | 4.2 | 0 | 0 | 100 |
| 07. Dec 20 | 95.3 | 0 | 0 | 4.7 | 0 | 0 | 100 |
| 14. Dec 20 | 94.9 | 0 | 0 | 5.1 | 0 | 0 | 100 |
| 21. Dec 20 | 94.3 | 0.09 | 0 | 5.6 | 0.01 | 0 | 100 |
| 28. Dec 20 | 93.26 | 0.76 | 0 | 5.94 | 0.04 | 0 | 100 |
| 04. Jan 21 | 92.4 | 1.08 | 0 | 6.45 | 0.07 | 0 | 100 |
| 11. Jan 21 | 91.5 | 1.44 | 0 | 6.96 | 0.09 | 0 | 100 |
| 18. Jan 21 | 90.11 | 2.12 | 0.13 | 7.49 | 0.14 | 0.01 | 100 |
| 25. Jan 21 | 88.62 | 2.67 | 0.66 | 7.82 | 0.2 | 0.04 | 100 |
| 01. Feb 21 | 87.16 | 3.28 | 1.09 | 8.14 | 0.26 | 0.08 | 100 |
| 08. Feb 21 | 85.71 | 3.6 | 1.8 | 8.45 | 0.3 | 0.13 | 100 |
| 15. Feb 21 | 84.67 | 3.59 | 2.57 | 8.66 | 0.31 | 0.2 | 100 |
| 22. Feb 21 | 83.7 | 3.39 | 3.55 | 8.77 | 0.31 | 0.28 | 100 |
| 01. Mar 21 | 82.79 | 3.35 | 4.32 | 8.87 | 0.31 | 0.35 | 100 |
| 08. Mar 21 | 81.86 | 3.38 | 5.05 | 8.98 | 0.32 | 0.42 | 100 |
| 15. Mar 21 | 80.29 | 3.94 | 5.81 | 9.08 | 0.4 | 0.49 | 100 |
| 22. Mar. 21 | 77.83 | 5.06 | 6.72 | 9.27 | 0.54 | 0.58 | 100 |
| 29. Mar. 21 | 74.85 | 6.63 | 7.74 | 9.35 | 0.74 | 0.69 | 100 |
| 05. Apr 21 | 70.92 | 8.81 | 8.91 | 9.51 | 1.02 | 0.82 | 100 |
| 12. Apr 21 | 66.63 | 10.87 | 10.62 | 9.53 | 1.33 | 1.02 | 100 |
| 19. Apr 21 | 61.66 | 12.75 | 13.11 | 9.51 | 1.65 | 1.32 | 100 |
| 26. Apr 21 | 56.17 | 14.33 | 16.27 | 9.53 | 1.97 | 1.73 | 100 |
| 03. May 21 | 52.1 | 14.81 | 19.34 | 9.44 | 2.15 | 2.16 | 100 |
| 10. May 21 | 48.88 | 14.26 | 22.78 | 9.26 | 2.18 | 2.65 | 100 |
| 17. May 21 | 46.49 | 12.95 | 26.13 | 9.21 | 2.05 | 3.17 | 100 |
| 24. May 21 | 44.36 | 11.25 | 29.64 | 9.17 | 1.85 | 3.72 | 100 |
| 31. May 21 | 42.47 | 10.18 | 32.39 | 9.03 | 1.75 | 4.18 | 100 |
| 07. Jun 21 | 40.9 | 9.54 | 34.44 | 8.9 | 1.7 | 4.53 | 100 |
| 14. Jun 21 | 38.6 | 7.77 | 38.51 | 8.4 | 1.47 | 5.25 | 100 |
| 21. Jun 21 | 37.08 | 6.46 | 40.96 | 8.53 | 1.26 | 5.72 | 100 |
| 28. Jun 21 | 35.8 | 5.26 | 43.06 | 8.7 | 1.06 | 6.12 | 100 |
| 05. Jul 21 | 34.72 | 4.22 | 44.8 | 8.9 | 0.88 | 6.47 | 100 |
| 12. Jul 21 | 33.81 | 3.34 | 46.23 | 9.13 | 0.72 | 6.77 | 100 |
| 19. Jul 21 | 33.03 | 2.62 | 47.37 | 9.37 | 0.59 | 7.02 | 100 |
| 26. Jul 21 | 32.19 | 2.2 | 48.28 | 9.59 | 0.52 | 7.22 | 100 |
| 02. Aug 21 | 31.44 | 1.89 | 48.99 | 9.82 | 0.47 | 7.39 | 100 |
| 09. Aug 21 | 30.75 | 1.69 | 49.54 | 10.05 | 0.44 | 7.53 | 100 |
| 16. Aug 21 | 30.12 | 1.57 | 49.96 | 10.28 | 0.43 | 7.64 | 100 |
| 23. Aug 21 | 29.54 | 1.34 | 50.45 | 10.52 | 0.39 | 7.77 | 100 |
| 30. Aug 21 | 28.99 | 1.17 | 50.86 | 10.75 | 0.35 | 7.89 | 100 |
| 06. Sep 21 | 28.46 | 1.03 | 51.21 | 10.98 | 0.33 | 7.99 | 100 |
| 13. Sep 21 | 27.96 | 0.93 | 51.51 | 11.21 | 0.31 | 8.09 | 100 |
| 20. Sep 21 | 27.49 | 0.84 | 51.77 | 11.43 | 0.29 | 8.18 | 100 |
| 27. Sep 21 | 27.03 | 0.78 | 52.01 | 11.65 | 0.28 | 8.26 | 100 |
| 04. Oct. 21 | 26.58 | 0.73 | 52.22 | 11.85 | 0.28 | 8.33 | 100 |
| 11. Oct. 21 | 26.15 | 0.69 | 52.42 | 12.05 | 0.27 | 8.41 | 100 |
| 18. Oct. 21 | 25.74 | 0.66 | 52.6 | 12.25 | 0.27 | 8.48 | 100 |
| 25. Oct. 21 | 25.33 | 0.64 | 52.77 | 12.43 | 0.27 | 8.55 | 100 |
| 01. Nov 21 | 24.94 | 0.62 | 52.94 | 12.61 | 0.27 | 8.62 | 100 |
| 08. Nov 21 | 24.56 | 0.6 | 53.1 | 12.78 | 0.27 | 8.69 | 100 |
| 15. Nov 21 | 24.18 | 0.59 | 53.25 | 12.94 | 0.28 | 8.76 | 100 |
| 22. Nov 21 | 23.82 | 0.57 | 53.4 | 13.09 | 0.28 | 8.83 | 100 |
| 29. Nov 21 | 23.46 | 0.56 | 53.55 | 13.24 | 0.28 | 8.9 | 100 |
| 06. Dec 21 | 23.12 | 0.56 | 53.69 | 13.37 | 0.29 | 8.97 | 100 |
| 13. Dec 21 | 22.78 | 0.55 | 53.83 | 13.5 | 0.29 | 9.05 | 100 |
| 20. Dec 21 | 22.45 | 0.54 | 53.97 | 13.62 | 0.3 | 9.12 | 100 |
| 27. Dec 21 | 22.13 | 0.53 | 54.1 | 13.73 | 0.3 | 9.19 | 100 |
| 03. Jan 22 | 21.82 | 0.53 | 54.24 | 13.84 | 0.3 | 9.27 | 100 |
| 10. Jan 22 | 21.52 | 0.52 | 54.37 | 13.93 | 0.31 | 9.35 | 100 |
| 17. Jan 22 | 21.22 | 0.52 | 54.5 | 14.02 | 0.31 | 9.42 | 100 |
| 24. Jan 22 | 20.93 | 0.51 | 54.63 | 14.11 | 0.32 | 9.5 | 100 |
| 31. Jan 22 | 20.65 | 0.51 | 54.76 | 14.18 | 0.32 | 9.58 | 100 |
| 07. Feb 22 | 20.37 | 0.5 | 54.89 | 14.25 | 0.32 | 9.66 | 100 |
| 14. Feb 22 | 20.1 | 0.5 | 55.01 | 14.31 | 0.33 | 9.74 | 100 |
| 21. Feb 22 | 19.84 | 0.5 | 55.14 | 14.37 | 0.33 | 9.83 | 100 |
| 28. Feb 22 | 19.59 | 0.49 | 55.26 | 14.42 | 0.34 | 9.91 | 100 |
| 07. Mar. 22 | 19.34 | 0.49 | 55.39 | 14.46 | 0.34 | 9.99 | 100 |
| 14. Mar. 22 | 19.09 | 0.48 | 55.51 | 14.5 | 0.34 | 10.08 | 100 |

CDC, Centres for Disease Control and Prevention
