# Supplementary material for: Modelling the concentration of anti-SARS-CoV-2 immunoglobulin G in intravenous immunoglobulin product batches
Source: PLoS One. 2021 Nov 29;16(11):e0259731. doi: 10.1371/journal.pone.0259731 (PMC8629175; doi:10.1371/journal.pone.0259731)
Supplement: S4 Table — (DOCX) [file pone.0259731.s004.docx]

*Modelling the concentration of anti-SARS-CoV-2 immunoglobulin G in intravenous immunoglobulin product batches.*

**Supplementary data**

**S4 Table. Observed EliA S1-IgG concentration for convalescent plasma pools (250 donations per pool).**

| **Pool #** | **Conc. S1-IgG (U/mL)** |
| --- | --- |
| 1 | 88.6 |
| 2 | 97.5 |
| 3 | 77.4 |
| 4 | 58.7 |
| 5 | 97.5 |
| 6 | 67.2 |
| Mean | 81.2 |

IgG, immunoglobulin G.
