# Supplementary material for: Modelling the concentration of anti-SARS-CoV-2 immunoglobulin G in intravenous immunoglobulin product batches
Source: PLoS One. 2021 Nov 29;16(11):e0259731. doi: 10.1371/journal.pone.0259731 (PMC8629175; doi:10.1371/journal.pone.0259731)
Supplement: S5 Table — (DOCX) [file pone.0259731.s005.docx]

*Modelling the concentration of anti-SARS-CoV-2 immunoglobulin G in intravenous immunoglobulin product batches.*

**Supplementary data**

**S5 Table. Privigen batches used in this study.**

| **Lot Number** | **Date of Manufacture** |
| --- | --- |
| P100233273 | 01.06.2020 |
| P100233274 | 02.06.2020 |
| P100233278 | 04.06.2020 |
| P100233279 | 05.06.2020 |
| P100233280 | 06.06.2020 |
| P100233281 | 07.06.2020 |
| P100256988 | 27.08.2020 |
| P100258461 | 07.09.2020 |
| P100258462 | 16.09.2020 |
| P100287013 | 25.11.2020 |
| P100287895 | 27.11.2020 |
| P100287898 | 28.11.2020 |
| P100287903 | 01.12.2020 |
| P100291363 | 05.12.2020 |
| P100313499 | 21.02.2021 |
| P100314413 | 23.02.2021 |
| P100313454 | 26.02.2021 |
| P100313455 | 02.03.2021 |
| P100314613 | 04.03.2021 |
| P100195250 | 05.03.2021 |
| P100314615 | 05.03.2021 |
| P100319628 | 20.03.2021 |
| P100326406 | 10.04.2021 |
| P100326433 | 10.04.2021 |
| P100326434 | 11.04.2021 |
| P100326411 | 11.04.2021 |
| P100326911 | 15.04.2021 |
| P100331025 | 25.04.2021 |
| P100334257 | 28.04.2021 |
| P100334353 | 28.04.2021 |
| P100334354 | 29.04.2021 |
| P100334323 | 02.05.2021 |
| P100326930 | 04.05.2021 |
| P100336740 | 05.05.2021 |
| P100336742 | 06.05.2021 |
| P100336751 | 06.05.2021 |
| P100339852 | 13.05.2021 |
| P100326941 | 19.05.2021 |
| P100240433 | 24.05.2021 |
| P100343169 | 28.05.2021 |
| P100347755 | 04.06.2021 |
| P100348971 | 12.06.2021 |
