# Supplementary material for: Modelling the concentration of anti-SARS-CoV-2 immunoglobulin G in intravenous immunoglobulin product batches
Source: PLoS One. 2021 Nov 29;16(11):e0259731. doi: 10.1371/journal.pone.0259731 (PMC8629175; doi:10.1371/journal.pone.0259731)
Supplement: S1 File — (DOCX) [file pone.0259731.s006.docx]

*Modelling the concentration of anti-SARS-CoV-2 immunoglobulin G in intravenous immunoglobulin product batches*

**Supplementary information**

**Statistical methods**

*The population model*

The proportions of the target population in each group and at each timepoint are derived from data published by the US CDC. Hereby, the CDC provides weekly information on the accumulated proportion of the target population with a current or previous COVID-19 infection ${NI}_{j}$, with at least one vaccination ${FV}_{j}$, and with two vaccinations ${SV}_{j}$. Hereby $j\geq1$ denotes the timepoint in weeks since the first COVID-19 infections were reported by the CDC, $i \in\{1,\ldots,6\}$ denotes the grouping according to infection status and number of vaccinations, and $p_{i,j}$ the proportion of the target population in each group $i$ at time point $j$. In this setting,

${NI}_{j}= \sum_{i\in\{4, 5,6\}} p_{i,j}, {FV}_{j}= \sum_{i\in\{2,3, 5,6\}} p_{i,j},$and ${SV}_{j}= \sum_{i\in\{3, 6\}} p_{i,j}$,

and following standard notations, we define the weekly change $\Delta$ in the respective quantities as ${\Delta NI}_{j}= {NI}_{j}{-NI}_{j-1}= \sum_{i\in\left\{ 4, 5,6 \right\}} \Delta p_{i,j}$, ${\Delta FV}_{j}= {FV}_{j}{-FV}_{j-1}= \sum_{i\in\{2,3, 5,6\}} {\Delta p}_{i,j}$, and ${\Delta SV}_{j}= {SV}_{j}{-SV}_{j-1}= \sum_{i\in\{3,6\}} \Delta p_{i,j}$ for $j\geq2$.

In view of the standard requirements for proportions, namely $0\leq p_{i,j}\leq1 \forall j$, $i$ $\in\left\{ 1,\ldots,6 \right\}$, $\sum_{i\in\left\{ 1,\ldots,6 \right\}} p_{i,j}=1 \forall j$ and $\sum_{i\in\left\{ 1,\ldots,6 \right\}} {\Delta p}_{i,j}=0 \forall j$, one additional assumption is required to derive all proportions. As the first and second vaccinations were provided to the target population without any differentiation based on the infection status, we assume that the vaccinations in each week were split proportionally according to the proportions of naïve and infected donors without that vaccination in the previous week. We illustrate this assumption as well as the computation exemplarily for an arbitrary week and denote this week as $t$. Clearly, for week $t-1$ the composition of the target population is known and ${\Delta SV}_{t}={\Delta p}_{3,t}+{\Delta p}_{6,t}$. Our assumption implies that${{\Delta p}_{3,t} / \Delta SV}_{t}= p_{2,t}$ / ($p_{2,t}+ p_{5,t}$) and allows to compute the transitions${\Delta p}_{3,t}$ and${\Delta p}_{6,t}$. With the same approach, the transitions ${\Delta p}_{2,t}$ and${\Delta p}_{3,t}$ can be computed from the change in first-time vaccinations, that is ${\Delta FV}_{t}-{\Delta SV}_{t}={\Delta p}_{2,t}+{\Delta p}_{5,t}$. Invoking ${\Delta NI}_{j}= \sum_{i\in\left\{ 4, 5,6 \right\}} \Delta p_{i,j}$ allows to compute $\Delta p_{4,j}$ and finally $\Delta p_{1,j}$ as the sum of the transitions in any given week is zero. Consequently, all transitions in week $j$ are known and all proportions $p_{i,j}$ can be derived. The final step is now to derive the residence time distribution, i.e., the proportions $p_{i,j,k}$ for all $k$, in each group and for each week. Based on the previously computed history of transitions between the groups and the obvious condition $\sum_{k} p_{i,j,k}=p_{i,j} \forall j$, $i$ $\in\left\{ 1,\ldots,6 \right\}$, computing the proportions $p_{i,j,k}$ is straight forward.

For the prediction of the proportions beginning from date of publication until March 2022 we extrapolate the ${NI}_{j}$, ${FV}_{j}$, and ${SV}_{j}$ curves as follows. Given that the occurrence and timing of future 4^th^, 5^th^, etc. COVID waves is hard to model, we rely on a Markov process for the weekly changes in ${NI}_{j}$ and adjust the transition probabilities over time such that lower weekly changes in ${NI}_{j}$ become more and more likely. Given the strongly symmetric evolution of ${FV}_{j}$ and ${\Delta FV}_{j}$ in combination with the well-known decrease in first vaccinations over the last weeks, we extrapolate ${FV}_{j}$ symmetrically and thus let ${\Delta FV}_{j}$ decay over the next ~3 months to a marginal level of new weekly first-time vaccinations. The proportion of second time vaccinated in the target population ${SV}_{j}$ follows the evolution of ${FV}_{j}$ with a lag phase of approximately four weeks at a slightly lower level, and we assume for the extrapolation of ${SV}_{j}$ that this relationship continues to hold. From this set of quantities, we then derive the proportions $p_{i,j,k}$ in the same way as previously described all data derived from these calculations are shown in S3 Table.

*Titre model*

The titre model specifies the average anti-SARS-CoV-2 titre $t_{i,k}$ for donors in group $i$ after $k$ weeks residence time. Hereby, an exponential decay was assumed after the initial transition phase of three weeks. As such, we rely on the titre values stated in S1 Table for the first three weeks and use from week 4 onwards the exponential decay model $t_{ik}=a_{i} exp(-\lambda_{i} (k-3))$, where $a_{i}$ is the titre value stated for week 3 and group $i$ in S1 Table, and $\lambda_{i}$ is the weekly decay rate that relates to the half-life time $t_{\frac{1}{2},i}$ stated in S2 Table via $\lambda_{i}=\ln\left( 2 \right)/ (t_{\frac{1}{2},i}/7)$.

Based on the population and titre model, the predicted anti-SARS-CoV-2 titre $T_{j}$ for plasma donated by the target population in week $j$ is the weighted titre average that invokes the proportions, residence time, and titre differences across the six groups.

$$T_{j}=\sum_{i=1}^{6} \sum_{k\geq0} p_{i,j,k}\cdot t_{ik},$$
